# Host Cell Virus Interactions: Molecular Mechanisms, Immune Modulation, Viral Pathogenesis, and Emerging Therapeutic Targets

**DOI:** 10.3390/v18010125

**Published:** 2026-01-18

**Authors:** Awadh Alanazi, Mohamed N. Ibrahim, Eman Fawzy El Azab, Mohamed A. Elithy

**Affiliations:** 1 Department of Clinical Laboratory Sciences, College of Applied Medical Sciences, Jouf University, Sakaka 72388, Saudi Arabia; 2 Department of Clinical Laboratories Sciences, College of Applied Medical Sciences at Al Qurayyat, Jouf University, Al Qurayyat 77454, Saudi Arabia; mnabil@ju.edu.sa (M.N.I.); efelazab@ju.edu.sa (E.F.E.A.); 3 Department of Biochemistry, Ain Shams University, Cairo 11544, Egypt

**Keywords:** antiviral treatments, functional genomic, host-directed antivirals, host–virus interactions, immune evasion, immune response, interferon signaling

## Abstract

Host–virus relationships regulate every phase of viral infection and critically influence course of illness and the effectiveness of treatment. Viruses utilize host receptors, intracellular trafficking routes, metabolic programs, and immunological signaling networks to introduce infection, while host cells use innate and adaptive immune responses that both limit viral replication and, in certain situations, cause tissue damage. Given the fast viral evolution and drug resistance linked to virus-directed therapy, there is growing proof that these host-dependent mechanisms are appealing and underutilized targets for antiviral treatment. Recent developments in single-cell technology, proteomics, and functional genomics have made it possible to systematically identify host dependency and restriction factors shared by different viral families, exposing common molecular vulnerabilities that might be targeted therapeutically. This review integrates current knowledge of virus–host interplay via a translational lens, highlighting processes that directly guide the formation of host-directed antivirals and immune-regulating treatments. We emphasize host processes involved in viral entry, replication, and immune signaling that have shown therapeutic significance, while illustrating the difficulties of balancing antiviral effectiveness with immunopathology. By framing host–virus interactions through a therapeutic lens, this review attempts to offer a targeted and translationally relevant viewpoint for next-generation antiviral research.

## 1. Introduction

Viral infections tend to pose major threats to world health and have influenced human history on multiple occasions. The 1918 influenza pandemic and the most recent COVID-19 pandemic have demonstrated how quickly viruses can spread and upend communities, leading to significant disease, fatalities, and unstable economies [1]. Beyond significant outbreaks, a lot of viral infections linger for years or cause cancer, which puts a continuous strain on healthcare systems around the globe. In spite of decades of antiviral studies and drug development, successful and lasting treatment options remain few for several viral infections, highlighting the necessity for new conceptual strategies to antiviral treatment.

A defining attribute of viral infection is the complete reliance of viruses on host cells for replication. Viruses are obligatory intracellular pathogens that cannot reproduce on their own due to a shortage of metabolic and biosynthetic resources. Viruses take advantage of host cellular machinery and signaling routes at each phase of the life cycle, including attachment, entrance, genome replication, protein synthesis, assembly, and release [2]. Important features of infection, such as viral tropism, replication efficiency, immune identification, and extent of illness, are determined by these virus–host relationships. These connections also produce exploitable vulnerabilities that are becoming more widely acknowledged as therapeutic potential since viral replication cannot persist without host assistance [3]. Accordingly, this review concentrates mainly on SARS-CoV-2, HIV-1, hepatitis C virus (HCV), and influenza A virus (IAV), which collectively offer well-researched illustrations of how RNA viruses propagate, defy host immunity, and respond to antiviral treatments.

In the past, the majority of antiviral treatments were developed to specifically target viral proteins including polymerases, proteases, and structural elements. Significant therapeutic successes have been achieved from this virus-centric strategy, especially in the therapy of HIV and HCV [4]. However, the swift mutation rates and genetic plasticity of several viruses, which enable quick adaptation under selective drug pressure, severely limit the efficacy of virus-directed therapeutics. Antiviral resistance thus often develops, requiring combination regimens and ongoing medication development. These restrictions have led to a rise in enthusiasm for host-directed antiviral tactics, which try to disrupt cellular processes necessary for virus replication or alter host reactions that affect the course of illness [5].

Because host components are biologically stable and frequently conserved across many virus families, host-directed antiviral strategies are especially attractive. Therefore, compared to virus-specific medications, attacking shared host dependence pathways may provide more antiviral effectiveness and a stronger barrier to resistance. Within this context, viral entry has proven to be one of the most translationally effective targets among the several stages of infection. Certain host receptors, co-receptors, and proteases are necessary for numerous viruses to enter target cells, which makes them appealing targets for treatment [6]. Clear evidence that host-directed methods can produce long-lasting antiviral efficacy is provided by clinical efficacy of inhibiting host entrance factors, such as CCR5 in HIV infection. In a similar vein, host proteases engaged in viral glycoprotein activation, such as those needed for SARS-CoV-2 entry through ACE2, have been determined as potential targets for respiratory viruses.

Viruses significantly alter host intracellular pathways after invasion in order to foster an environment that is conducive to reproduction. Different virus families frequently take over host functions related to vesicular trafficking, lipid metabolism, membrane remodeling, RNA processing, and protein quality control. The systematic discovery of host dependency variables necessary for viral replication has been made possible by developments in functional genomics, namely genome-wide CRISPR-Cas screening techniques [7]. Conserved host pathways that make appealing options for the creation of broad-spectrum antivirals have been identified by these investigations. Significantly, a large number of these host factors, such as kinases and metabolic enzymes, are already pharmacologically tractable, opening up possibilities for drug repurposing and quickening the translation process [8].

Apart from host dependence factors, immune system reactions are crucial in determining the course of infection. Early antiviral defense depends on innate immune sensing routes and interferon signaling, although immune activation is not always advantageous and can exacerbate disease pathology when it is excessive, protracted, or timed incorrectly [9]. This dual, or “double-edged,” nature of interferon signaling poses a significant obstacle to antiviral treatment. Recent data from COVID-19 and clinical successes with interferon-based therapies for viral hepatitis highlight how crucial timing, tissue context, and host immune status are to therapeutic success. These findings emphasize the necessity of striking a balance between preventing collateral tissue damage and antiviral immune stimulation [10].

The research on virus–host interactions and their translational potential has been greatly improved by new technological advancements. These days, high-throughput functional genomics, proteomics, single-cell transcriptomics, and systems biology techniques allow for the precise mapping of host pathways involved in infection while capturing variability across cell types and tissues [11]. These instruments have shown that a complicated interaction between viral variables, host genetics, immunological state, and cellular environment shapes infection outcomes. The logical development of host-directed antivirals and immune-modulating treatments with enhanced safety and effectiveness profiles is progressively being guided by these discoveries.

In this review, we concentrate on virus–host interplay as a basis for determining new therapeutic targets and translational approaches. We focus on host mechanisms involved in viral entry, replication, and immune control that are directly related to the development of antiviral drugs. This review attempts to offer a cogent paradigm for utilizing virus–host connections to create widely effective and resistance-resistant antiviral medicines by fusing molecular insights with translational and therapeutic concerns.

## 2. Molecular Mechanisms of Virus–Host Interaction

### 2.1. Viral Attachment and Entry

Viral invasion into host cells is a multi-part process that starts with attachment to cell surface receptors and ends in transfer of the viral genome into the cytoplasm or nucleus. Each stage includes specialized virus–host molecular connections that influence tissue tropism, host range, and sensitivity to restriction. Viruses begins infection via interplay between viral attachment proteins and host cell surface receptors or attachment factors, usually proteins or glycans with regular physiological functions [12]. One important aspect influencing which cells and tissues a virus may invade is the unique nature of interactions among viral surface proteins and host receptors. For instance, the spike protein of SARS-CoV-2 binds to angiotensin-converting enzyme 2 (ACE2) to gain entrance. The physiologic basis for the multi-organ engagement seen in COVID-19 is provided by the existence of ACE2 in the respiratory tract, gastrointestinal epithelium, and vascular endothelium. Furthermore, natural variations in ACE2 structure can change binding efficiency, which contributes to the understanding of how illness severity differs among people. Regarding influenza A virus, tropism is regulated by the receptor selectivity of hemagglutinin for sialic acid–possessing glycans. Human-adapted strains typically attach α2,6-linked sialic acids, which are plentiful in the human upper airway, while avian strains choose α2,3-linked sialic acids, which sets species barriers and restricts the effectiveness of human infection. HIV entrance is significantly more strictly controlled, requiring attachment to the chemokine co-receptors CCR5 or CXCR4 after a first encounter between gp120 and CD4. This periodic receptor engagement limits HIV infection to particular immune cell populations and significantly affects the spread of the virus and the course of the illness. The hepatitis C virus utilizes a variety of attachment and entry characteristics to explain stringent hepatotropism and guide antiviral entry-targeted treatments [13,14].

To enter host cells, numerous viruses need more than one receptor; instead, they rely on co-receptors, supplementary host proteins, and host proteases, all of which influence tissue tropism and entrance efficiency. One of the most crucial of these is the proteolytic activation of viral fusion proteins. In coronaviruses, host proteases such furin, TMPRSS2, or endosomal cathepsins must break the spike protein at the S1/S2 and S2′ locations. Whether spike activation happens during viral assembly, at the cell surface, or after endocytosis depends on the protease that is accessible, which affects the entrance process. In SARS-CoV-2 infection, TMPRSS2-driven activation at the cell surface facilitates quick fusion with the plasma membrane and is more effective than cathepsin-dependent endosomal invasion. Therefore, increasing cellular vulnerability is linked to greater TMPRSS2 expression, and inhibitors like camostat and nafamostat can prevent viral infection in vitro. Influenza virus also need protease cleavage of hemagglutinin, with restricted versus widespread protease use altering tissue tropism and virulence [13,15].

Once receptors are activated and viral proteins are properly engaged by host proteases, viruses gain entry to host cells either via immediate membrane fusion or through receptor-driven endocytosis, with fusion happening at the plasma membrane or within endosomes. The host cell type, host parameters, and viral properties all influence the particular entry mechanism that is employed [12]. Coronaviruses, influenza viruses, and HIV are examples of enveloped viruses that rely on fusion proteins that modify their shape in the wake of endosomal acidification, proteolytic processing, or receptor interaction. When TMPRSS2 exists, SARS-CoV-2 can fuse at the cell surface; otherwise, it can fuse inside endosomes after being activated by cathepsins. Adenoviruses and other non-enveloped viruses, on the other hand, enter through endocytosis and leave by rupturing endosomal membranes. Access is controlled by host endosomal trafficking machinery, which includes lipid kinases, ESCRT complexes, and Rab GTPases. CRISPR screens revealed PIKfyve and lipid metabolism processes as crucial coronavirus entrance regulators and possible treatment targets [13].

Proteases, host receptors, co-receptors, and intracellular elements necessary for replication work together to shape viral tropism. In many cases, successful infection requires more than just receptor expression. Primary lung infection in SARS-CoV-2 is caused by strong co-expression of ACE2 and TMPRSS2 in respiratory epithelium, whereas gastrointestinal, vascular, and renal involvement are explained by ACE2 expression in enterocytes, endothelium cells, and renal tubules. CCR5 or CXCR4 use and CD4 density influence the tropism of CD4 T cells or macrophages in HIV, which affects tissue reservoirs, disease course, and transmission routes [16]. As the initial line of antiviral defense, host cells generate intrinsic restriction factors that prevent viral entrance and early replication in addition to entry factors. By preventing virus–endosome fusion, interferon-induced transmembrane proteins (IFITM1–3) limit the penetration of numerous enveloped viruses, such as influenza and coronaviruses; IFITM3 mutations are associated with severe influenza [17]. Viral antagonists like HIV Vpu and SARS-CoV ORF7a are prompted by tetherin (BST-2), which restricts enveloped virus discharge and can affect entrance. SERINC3 and SERINC5 hinder the entrance of HIV through integration into virions, an effect that is countered by HIV Nef.

### 2.2. Viral Replication and Protein Synthesis

Viruses need to replicate their genetic material and employ the machinery of the host cell to make viral proteins after successfully entering and uncoating. This stage of infection is a crucial vulnerability that can be addressed therapeutically and entails numerous interactions between the virus and the host. In order to maximize viral gene expression, DNA viruses often reproduce in the nucleus by taking use of host DNA polymerases, transcription factors, and RNA processing machinery. They also actively manipulate cell cycle checkpoints and transcriptional programs. On the other hand, the majority of RNA viruses use viral RNA-dependent RNA polymerases to replicate in the cytoplasm, although they still rely heavily on the translation machinery of the host. Since viral mRNAs have to contend with host transcripts, several RNA viruses specifically hinder host translation. By cleaving initiation factors like eIF4G, picornaviruses and caliciviruses employ internal ribosome entrance sites to prevent cap-dependent host translation. Cap-snatching is a strategy used by the influenza virus to stimulate viral mRNAs and suppress host gene expression. Coronaviruses keep their RNAs capped and employ proteins like SARS-CoV-2 Nsp1 to prevent translation in hosts. Viral RNA stability and replication are additionally controlled by host RNA-binding proteins, such as proviral DDX3 and antiviral ZAP [13,15]. Numerous RNA viruses create specific membrane replication organelles that operate as hubs for the production of viral RNA, concentrating necessary components while preventing immune detection of viral RNA [18]. To create these frameworks, positive-strand RNA viruses alter host membranes from the ER, Golgi, or endosomes. While flaviviruses produce ER invaginations powered by nonstructural proteins like NS4A and NS4B, coronaviruses construct double-membrane vesicles [19]. This mechanism revolves around host lipid metabolism, with phospholipid, fatty acid, and cholesterol processes consistently found to be critical for effective viral replication [20].

Host endomembrane systems facilitate several phases of viral replication in addition to replication organelles. With the aid of chaperones, the ER facilitates the production and correct folding of viral glycoproteins, and ER stress and ERAD systems are frequently appropriated to process viral proteins or weaken host defenses [21]. The Golgi is a budding location for many enveloped viruses and changes viral proteins by furin cleavage and glycosylation. Viral assembly, transport, and egress are also facilitated by Rab-regulated trafficking and endosomal compartments [22]. Since viral replication is energy-intensive, several viruses actively alter host cellular metabolism. Especially, enveloped viruses increase lipid and cholesterol biosynthesis to facilitate membrane formation and the creation of replication organelles; for instance, HCV stimulates lipid synthesis via engaging sterol regulatory element–binding proteins (SREBPs), whereas SARS-CoV-2 relies on host cholesterol regulators such as HMGCR and NPC1. In order to meet biosynthesis and energetic requirements, numerous viruses, such as the dengue virus, also increase glycolysis and glutaminolysis [23]. Both host and viral variables influence the stability of viral RNA. Antiviral proteins like ZAP identify CpG-rich regions and encourage destruction, while host RNA decay mechanisms like NMD and OAS–RNase L attack viral RNAs. Viruses utilize structured UTRs, poly(A) tails, 5′ caps, and host RNA-binding proteins to defend their RNAs against this. In addition to viral polymerases, host helicases, kinases, and RNA-binding proteins—potential targets for antiviral therapy—are necessary for viral RNA production [24]. (Table 1).

### 2.3. Virus Assembly and Release

Progeny virions are assembled and released from infected cells during the last phases of the viral replication cycle. Viral structural proteins, genomes, and host trafficking machinery must all work together to facilitate these operations.

Viral assembly takes place at particular intracellular sites determined by targeting signals in viral structural proteins and the accessibility of suitable host membranes, such as the plasma membrane, ER, Golgi, and endosomes. Nucleocapsid or core assembly usually comes before envelope acquisition. In HIV, host ubiquitin ligases control Gag trafficking and assembly, whereas the Gag polyprotein promotes assembly at the plasma membrane by attracting viral RNA along with other components. Coronaviruses assemble at the ER–Golgi intermediate compartment, where M and E proteins facilitate budding and N packages genomic RNA are transported via the secretion route for discharge [11]. Correct transport of viral proteins to assembly sites depends on host trafficking systems. Viral glycoproteins created in the ER are transported via the Golgi, oftenhijacking COPI and COPII vesicles. Rab GTPases regulate vesicle movement, while motor proteins such as dynein and kinesin deliver viral components along microtubules, as noticed in adenovirus nuclear targeting and herpesvirus mobility to the cell surface [30].

Enveloped viruses obtain their lipid envelope by budding via the host cell membranes, this process frequently lies on the host ESCRT machinery, which regularly work in multivesicular body formation and cytokinesis. HIV and other retroviruses engage ESCRT via late-domain motifs (PTAP, PPXY, YPXL) in the Gag protein that attach adaptor proteins TSG101 and ALIX, resulting in ESCRT-III- driven membrane scission [31]. Immature virions are caught at the plasma membrane when ESCRT is disrupted. While some enclosed viruses, like the influenza virus, bud independently utilizing the intrinsic membrane scission ability of the M2 protein, plenty of other viruses, such as arenaviruses, filoviruses, and rhabdoviruses, also rely on ESCRT. Following budding, viruses go through proteolytic maturation, HIV protease cleavage of Gag and Gag-Pol is crucial for infectivity and a major target for treatment [32].

Several viruses move directly across cells by membrane nanotubes, virological synapses, or virus-induced cell–cell fusion in along with cell-free transmission. These methods are frequently more effective and aid in avoiding neutralizing antibodies [33]. While HTLV-1 employs comparable synapses and cellular conduits, HIV creates virological synapses among infected and uninfected T cells, concentrating the virus at the interaction site by host adhesion molecules and cytoskeletal rearrangement [16]. Spike-mediated syncytia generation is induced by SARS-CoV-2 and other coronaviruses, which facilitates the immediate cytoplasmic transport of viral components and contributes to the pathogenesis of COVID-19. Attacking ESCRT components, membrane trafficking routes, or fusion pathways along with direct-acting antivirals are examples of additional treatment options that arise from uncovering host variables related to assembly, budding, and cell-to-cell dissemination.

## 3. Host Immune Recognition and Viral Immune Evasion

### 3.1. Innate Immune Sensing

The initial line of defense toward viral infection is the innate immune system, which may identify viral components and start antiviral reactions within hours after infection. Pattern recognition receptors (PRRs), which identify pathogen-associated molecular patterns (PAMPs), such as viral proteins and nucleic acids, facilitate this quick reaction.

To detect viral infection in various compartments, cells employ a number of pattern recognition receptors. Viral RNA is detected in the cytoplasm by RIG-I–like receptors RIG-I (DDX58) and MDA5 (IFIH1). RIG-I detects 5′-triphosphate RNA and short double-stranded RNA, while MDA5 senses long double-stranded RNA. Each of them communicate via the adaptor MAVS on peroxisomal and mitochondrial membranes once they are activated. Viral nucleic acids are detected by toll-like receptors on the cell surface or inside endosomes, such as TLR3 for dsRNA, TLR7 and TLR8 for ssRNA, and TLR9 for CpG-rich DNA. These receptors communicate via MyD88 or TRIF. The cGAS–STING system detects cytoplasmic DNA from DNA viruses or retroviral intermediates, and cGAS produces cGAMP to stimulate STING and promote the synthesis of interferon [10].

The transcription factors that initiate the antiviral action, IRF3, IRF7, and NF-κB, are activated when pattern recognition receptors identify viral material. Kinases TBK1 and IKKε are activated by adaptor proteins such MAVS, TRIF, and STING, which causes IRF3 and IRF7 to be phosphorylated and enter the nucleus. In addition to inflammatory cytokines, this causes the production of type I interferons (IFN-α/β) and type III interferons (IFN-λ). Through the IFNAR receptor, type I interferons then affect identical and nearby cells, triggering JAK–STAT signaling and creating the ISGF3 complex, which promotes the production of interferon-stimulated genes. Type III interferons are crucial for antiviral protection at epithelial surfaces because they communicate via a different receptor but engage the same cascade [34].

Interferon signaling drives a wide antiviral state via the concerted expression of hundreds of interferon-stimulated genes (ISGs), which collectively limit viral replication at almost every phase of the viral life cycle. ISGs operate as a cohesive network to restrict viral entrance, genome replication, protein synthesis, assembly, and release instead of operating independently. Inhibition of viral translation, destruction of viral RNA, disruption of membrane fusion, and improvement of infected cell clearance are examples of well-established effector mechanisms [35]. Classical antiviral effectors involve RNA-centered processes including PKR-driven translational arrest and the OAS–RNase L system, as well as membrane-related restriction factors that hinder viral entrance or egress. Other ISGs alter innate immune signaling thresholds, lipid metabolism, and intracellular trafficking, changing the intracellular milieu to make it less conducive to viral multiplication. Crucially, these responses’ antiviral efficacy results from their synchronized and cumulative engagement rather than from any one ISG operating in isolation [36].

Few ISGs have been effectively used as primary therapeutic agents, despite the fact that they have been well studied in experimental systems. Rather, their main therapeutic significance is in determining the amount, timing, and tissue specificity of interferon responses, which ultimately dictate whether viral management is attained with minimum tissue damage or advances toward immunopathology. Genetic diversity in interferon signaling elements and ISG regulatory areas additionally influence interindividual variations in viral susceptibility and extent of illness [37].

Thus, interferon signaling is an antiviral process with two sides. Effective immune priming and quick viral suppression depend on early and properly controlled interferon responses. On the other hand, prolonged, excessive, or delayed interferon stimulation can worsen tissue healing, intensify inflammatory cascades, and significantly increase immunopathology. This equilibrium is especially noticeable in chronic viral illnesses and respiratory viral infections, when delayed interferon responses are linked to serious illness consequences [36]. As a result, the exact timing of use is crucial to the therapeutic effectiveness of interferon-based or interferon-modulating treatments.

### 3.2. Viral Strategies to Evade Innate Immunity

To prevent or inhibit innate immune responses, viruses have developed sophisticated tactics. These evasion methods play a significant role in pathogenesis and are essential virulence factors. Numerous viruses effectively disrupt pattern-recognition receptor signaling in order to thwart the interferon response. Targeting MAVS, the essential adapter for RIG-I-like receptors, is a popular tactic. While the hepatitis A virus’s 3C protease interferes with both MAVS and TRIF, hence blocking antiviral signaling, the hepatitis C virus utilizes its NS3/4A protease to cleave MAVS. Other viruses work downstream by blocking the kinases TBK1 and IKKε, which are necessary for IRF3 and IRF7 stimulation. For instance, dengue virus NS2B/3 cleaves STING to prevent DNA sensing, whereas herpes simplex virus ICP0 encourages TBK1 breakdown. Additionally, certain viruses, like the Ebola virus VP35, conceal their nucleic acids in membrane-bound replication compartments or generate decoy RNAs that engage PRRs without initiating signaling. In order to widely reduce interferon induction, SARS-CoV-2 employs a variety of strategies, including the production of minimal dsRNA and the deployment of Nsp1, Nsp3, Nsp6, and ORF6 [38].

Numerous viruses block ISG expression and maintain replication by interfering with downstream signaling, even when interferons are generated. The JAK–STAT system is a common target, paramyxovirus V proteins engage both STAT1 and STAT2, influenza virus NS1 blocks STAT1 phosphorylation, and dengue virus NS5 promotes STAT2 disintegration. Additionally, certain viruses degrade antiviral responses by decreasing the expression of interferon receptors or directly opposing ISGs such PKR, APOBEC3, and tetherin [39]. (Table 2).

**Figure 1:**
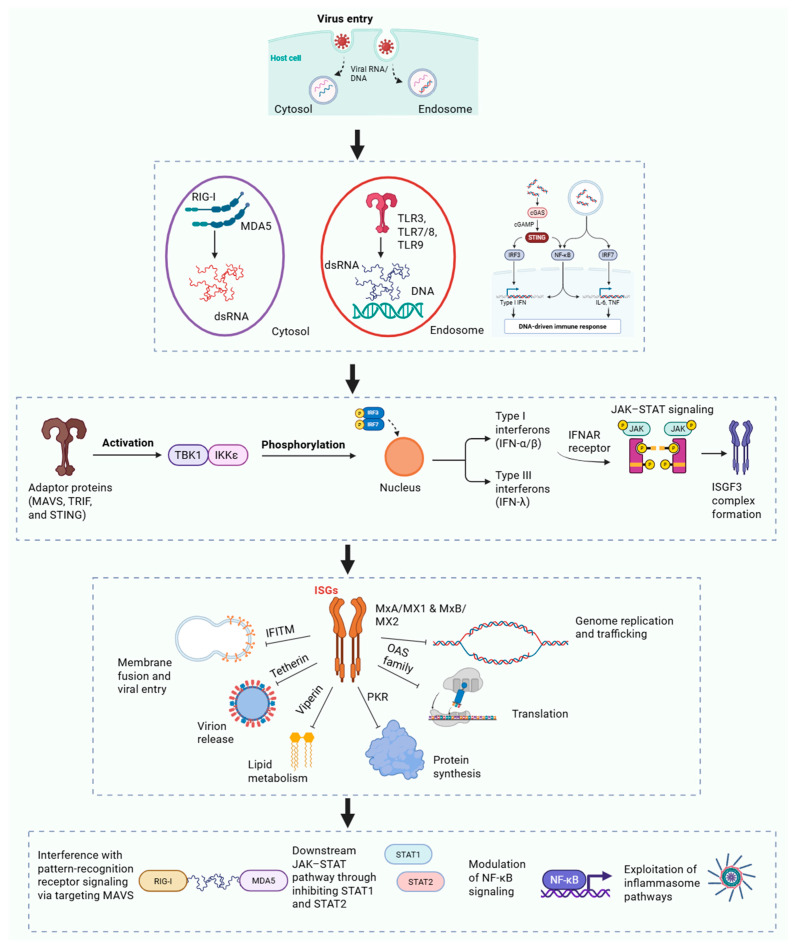
Innate Immune Identification of Viruses and Associated Viral Evasion Strategies.

In addition to interferon reactions, viruses alter other immune system processes. Viruses frequently target NF-κB signaling, either stimulating it to promote cell survival and replication or decreasing it to reduce inflammation. Similarly, pyroptosis-causing inflammasome processes that produce IL-1β and IL-18 are either blocked or used to promote viral propagation [40]. The progress of a viral infection is determined by how successfully the body strikes a balance between inflammation and antiviral responses. By attracting immune cells, inflammation aids in the management of infections, but when it gets out of control, it harms tissues and exacerbates illness. While some viruses cause an excessive production of cytokines, others reduce inflammation to ensure long-term survival. This unchecked reaction causes ARDS and multi-organ failure during serious influenza and COVID-19, underscoring the necessity of properly timed immune-modulating therapies. (Figure 1).

**Table 2:** Immune Modulation and Viral Pathogenesis.

Virus	Immune Pathway Targeted	Viral Immune Modulation Strategy	Pathogenic Consequence	References
SARS-CoV-2	Type I and III interferon responses	Several molecules of innate antiviral signaling systems are targeted by the viral genome.	Enhanced viral replication and serious illness	[41]
HCV	RIG-I–MAVS signaling	MAVS adaptor protein is cleaved by NS3/4A protease.	Inhibition of innate immune stimulation	[42]
Herpesviruses (HCMV)	Cytokine and chemokine signaling	Viral cytokine and chemokine homologs expressed	Immune escape and latency preservation	[43]
HIV-1	MHC-I antigen presentation	MHC-I molecules are internalized and degraded by Nef.	Evasion from cytotoxic T-cell–induced death	[8]
HBV	Type I interferon signaling	Viral proteins reduce the expression of ISG and STAT signals.	Development and retention of ongoing infections	[44]
Influenza A virus	Autophagy pathway	The viral M2 protein prevents the development of autophagosomes.	Increased viral replication and immune escape	[45]

### 3.3. Effects on Adaptive Immunity

While innate immunity offers instant resistance, adaptive immunity produces pathogen-specific reactions that offer long-term defense. Likewise, viruses encourage persistence and reinfection by interfering with adaptive immune system reactions. Viral peptides on MHC class I are necessary for CD8+ T cell identification, and several viruses avoid protection by interfering with this process. Viral peptides on MHC class I are essential for CD8+ T cell identification, and many viruses avoid protection by interfering with this process. Herpesviruses are very potent, with HCMV proteins US2, US3, US6, and US11 disintegrate, retain, or inhibit peptide loading onto MHC I, while HIV Nef lowers surface MHC I. EBV BZLF1 also blocks MHC class II expression, decreasing CD4+ T cell assistance [46]. Certain viruses effectively reduce MHC expression by suppressing transcription, encouraging breakdown, or trapping molecules within the cell, in addition to interacting with antigen processing. While KSHV K3 and K5 ubiquitinate MHC I for endocytosis and disintegration, adenovirus E3/19K keeps MHC class I in the ER. However, a few viruses generate MHC-like molecules that block NK cells while eluding T cells since decreased MHC I can cause NK cell death [47].

Adaptive immune regulation is additionally hampered by viral genetic variety in along with disruption of antigen presentation. Various RNA viruses produce heterogeneous viral populations that can swiftly choose escape variants since they reproduce with low fidelity. HIV is a prime example, where significant Env glycosylation obscures important antibody targets and mutations in Gag, Pol, and Env impair CD8^+^ T-cell recognition. Antigenic drift and sporadic shifts cause influenza to constantly mutate, which fuels recurring epidemics. SARS-CoV-2 has also developed spike mutations that lessen antibody neutralization in subsequent versions [48].

## 4. Role of Host Factors in Viral Pathogenesis

### 4.1. Cytopathic Effects and Cell Death

Numerous controlled pathways are used by viruses to cause host cell death, and the manner of cell death has a significant impact on inflammation, immunological responses, and the course of the disease. Apoptosis, necroptosis, and pyroptosis are the three main types that are pertinent to viral infections, each has unique biochemical and immunological ramifications.

The controlled, non-inflammatory process of apoptosis is marked by DNA fragmentation, cell shrinkage, and the creation of apoptotic bodies that are effectively removed by phagocytes. Apoptosis can be triggered by stress related to viral replication and, if triggered early, may serve as an antiviral response. Nevertheless, a number of viruses alter apoptotic routes, either by inducing apoptosis following replication to facilitate viral spread or by postponing cell death through viral Bcl-2 homologs [49].

Necroptosis is an orchestrated necrotic process driven by RIPK1, RIPK3, and MLKL, resulting in membrane rupture and inflammatory discharge of intracellular materials. When apoptosis is prevented, it frequently acts as a substitute, aiding in tissue pathology and virus prevention. Pyroptosis is a highly inflamed cell death caused by inflammasomes and facilitated by caspases and gasdermins, which releases IL-1β and IL-18. Extreme pyroptosis can cause immunopathology even though it is efficient at removing infected cells. The equilibrium between these pathways significantly influences both the pathogenesis of viruses and the results of treatment [50].

### 4.2. Inflammation and Tissue Injury

Although immune responses are crucial for eliminating viral infections, they can also be a significant cause of illness when they are overactive or improperly controlled, sometimes doing more harm than the virus itself. Thus, a key component of many viral diseases involves immune-mediated pathogenesis. Cytokine disruption is one of the most well-known mechanisms. The “cytokine storm,” characterized by higher levels of proinflammatory cytokines such as IL-6, TNF-α, and IL-1β, has been identified in serious outbreaks of influenza, SARS, MERS, and COVID-19. Positive feedback loops encompassing both innate and adaptive immune cells can cause these heightened reactions, which can result in vascular leakage, coagulopathy, systemic inflammation, acute respiratory distress syndrome, and multi-organ failure [51]. Although type I interferons are essential for antiviral protection, disease can also result from high or persistent interferon signaling. In HIV infection, immunological activation and CD4 T-cell depletion are associated with chronic interferon activation, and interferon signaling inhibition can occasionally enhance outcomes, according to experimental models. Migration of immune cells into infected tissues additionally worsens damage. Cytotoxic CD8^+^ T cells eradicate infected cells via perforin/granzyme and Fas-driven pathways, but this can compromise tissue integrity, especially in organs with reduced regenerative ability, such as the liver in hepatitis B and C [52]. Other immune-mediated mechanisms involve antibody-dependent enhancement, well described in dengue virus infection, and endothelial dysfunction with vascular leakage, microthrombosis, and coagulopathy, which are key characteristics of serious dengue and COVID-19.

### 4.3. Chronic Infection and Oncogenesis

Numerous viruses cause long-lasting infections that might endure for months or even a lifetime, drastically changing the physiology of the host and encouraging chronic illness. immuno evasion, latency, integration into the host genome, or infection of immuno-privileged areas all contribute to persistence, which causes persistent inflammation, immune fatigue, and increasing organ damage. Chronic liver infections are frequently caused by hepatitis B and C viruses, and years of constant inflammation is linked to cirrhosis, fibrosis, and hepatocellular cancer. The development of the disease is influenced by insertional mutagenesis (HBV), T-cell exhaustion, viral immune escape, and the immediate carcinogenic impacts of viral proteins [53]. HIV creates lifetime infection by entering into the host genome and builidng latent reservoirs in resting CD4^+^ T cells. Even under inhibitory antiretroviral treatment, prolonged immunological activation and inflammation raise the likelihood of cancer, neurocognitive decline, and cardiovascular disease. Herpesviruses survive through latency with occasional reactivation, herpes simplex virus and varicella-zoster virus stay dormant in neurons, while cytomegalovirus induces serious illness in immunocompromised hosts [54]. Viral oncogenesis, which accounts for about 15% of human malignancies, can result from persistent infection. By inactivating p53 and Rb through E6 and E7, high-risk HPV strains cause cervical and oropharyngeal malignancies. Viral oncoproteins, persistent inflammation, and immune bypass are ways that the Epstein–Barr virus, HBV, HCV, HTLV-1, and KSHV cause cancer. Curative antiviral drugs and preventive vaccinations demonstrate the medical value of emphasizing viral persistence [55].

## 5. Host-Targeted Antiviral Therapeutic Strategies

### 5.1. Targeting Viral Entry and Host Receptors

Preventing viral invasion is an appealing therapeutic approach because it stops infection before viral genome replication starts, possibly reducing tissue damage and immune activation. Entry antagonists might target host receptors, viral proteins, or the actual entry mechanism. Receptor inhibitors impede entry by blocking the virus’s ability to attach to cellular receptors. The CCR5 inhibitor maraviroc blocks HIV-1 entrance by attaching to the co-receptor and causing conformational modifications that stop envelope glycoprotein interaction. Maraviroc works well against CCR5-tropic HIV-1 strains and shows that host protein targeting can produce long-lasting antiviral effects with little development of resistance. Nevertheless, HIV-1 can use other co-receptors (CXCR4), which restricts the use of CCR5 blockers [56].

Another strategy is the utilization of soluble receptor decoys, which attach and kill viruses before they reach target cells by utilizing recombinant soluble forms of viral receptors. Soluble ACE2, which can neutralize SARS-CoV-2 by engaging the spike protein, has been investigated as a COVID-19 treatment. Because viruses cannot readily change receptor binding without decreasing infectivity, this strategy theoretically has a significant barrier to resistance [57]. Moreover, fusion inhibitors prevent viruses from entering through the membrane fusion stage. By attaching itself to HIV-1 gp41, the peptide fusion inhibitor enfuvirtide stops the conformational shifts necessary for membrane merging. Enfuvirtide is potent, but its use is limited because it needs to be injected subcutaneously and resistance can arise. Fusion inhibitors are being researched for additional viruses, such as respiratory syncytial virus and influenza [58].

**Figure 2:**
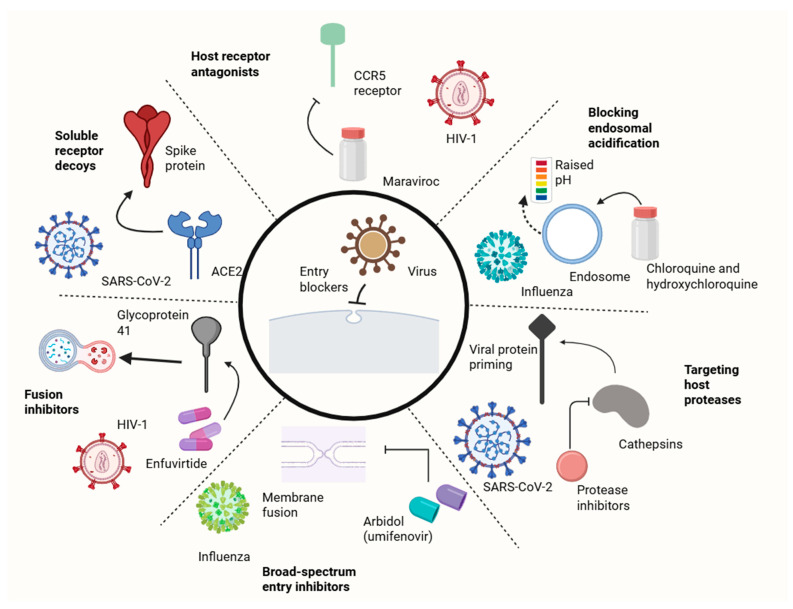
Host-targeted therapeutic approaches focusing on viral entry pathway.

Also, lysosomotropic drugs can prevent pH-dependent viral entrance by attacking endosomal acidification. By increasing endosomal pH, chloroquine and hydroxychloroquine prevent viruses like influenza and SARS-CoV-2 from fusing. Yet, clinical trials have not consistently shown efficacy for COVID-19; this could be because of alternate entry mechanisms or inadequate drug concentrations at pertinent areas [59]. Lastly, promising strategies for pandemic preparedness include broad-spectrum entry inhibitors which attack host mechanisms. Arbidol (umifenovir) has demonstrated effectiveness against influenza and other enveloped viruses and is thought to prevent membrane fusion by interacting with lipids. Another broad-spectrum strategy is the use of inhibitors of cathepsins and other endosomal proteases necessary for viral protein priming. However, considering the significance of these activities for regular cellular operations, creating such inhibitors with a suitable safety profile continues to be difficult [5,60]. (Figure 2).

### 5.2. Modulating Host Immune Responses

Interferon signaling regulation is a key but difficult component of host-directed antiviral therapies. Although interferons have a unique ability to induce broad-spectrum antiviral states, their pleiotropic influences on inflammation and tissue homeostasis limit their therapeutic value. Clinical experience with a variety of viral infections has shown that interferon signaling can be both protective and pathogenic, highlighting the fact that precise oversight of timing, dosage, and anatomical localization is more important for successful treatment than basic immune activation [61]. These issues are demonstrated by the utilization of interferon-α in chronic viral hepatitis in the past. Direct-acting antivirals eventually replaced interferon therapy due to its systemic toxicity and poor efficacy, even though it improved antiviral immunity and achieved viral suppression in a subgroup of patients. The focus has lately switched to type III interferons (IFN-λ), which have antiviral activities primarily at epithelial surfaces and reduce systemic inflammation, perhaps improving the treatment window [62].

Immune checkpoint drugs, which have transformed cancer treatment, are being investigated for persistent viral infections. In persistent infections, blocking PD-1 or PD-L1 can boost viral clearance by revitalizing worn-out T cells. Anti-PD-1 medication has showed promise in hepatitis B and HIV-positive patients, although cautious assessment is necessary due to worries about immune-related side effects and possible immune-driven tissue injury. The best benefit-risk profiles may be obtained by mixing checkpoint blockage with other antivirals [63].

Immunosuppressive treatments, on the other hand, might be helpful when immunopathology is the primary cause of illness. In individuals with severe COVID-19, corticosteroids have been proven to reduce inflammation and improve mortality, especially in those who need extra oxygen. However, immunosuppression increases the risk of secondary infections and prolongs viral replication, necessitating meticulous choice of patients and timing. JAK inhibitors and IL-6 receptor antagonists (tocilizumab) have also been investigated for COVID-19 with mixed outcomes, underscoring the difficulty of regulating immunity throughout viral infections [64,65]. The goal of prophylactic vaccination during ongoing infection is to increase antiviral immunity and encourage viral clearance. This strategy is being studied for HIV and chronic hepatitis B, with conflicting findings thus far. To combat established immune tolerance and viral persistence mechanisms, therapeutic vaccinations may need to be combined with additional immunomodulatory strategies or antivirals.

### 5.3. Host Dependency Factors as Drug Targets

Many possible treatment targets have been identified through the systematic identification of host factors necessary for viral replication. Numerous viruses’ host reliance variables have been mapped using genome-wide CRISPR and RNA interference screens, revealing both identified and unidentified targets. Kinase signaling, lipid metabolism, protein trafficking, and RNA processing are just a few of the various cellular processes that these variables affect. The importance of host kinase signaling pathways in viral replication is reflected in the growing emphasis on kinase antagonists in host-targeted antiviral therapies. Several viruses need particular host kinases to facilitate their life cycles, the dengue virus relies upon MTOR function to maintain effective replication, the hepatitis C virus needs PI4KIIIα to build replication complexes, and the influenza virus rely on PI3K signaling. Because of these common dependencies, host kinases are appealing targets for broad-spectrum antiviral therapy [66,67].

A fine example of this strategy is imatinib. This tyrosine kinase antagonist was first created to combat chronic myeloid leukemia. By blocking Abelson (Abl) kinase, it inhibits the replication of multiple viruses, notably poxviruses and SARS-CoV [68]. Building on these discoveries, many kinase inhibitors that have been approved for use in cancer treatments are being assessed for antiviral repurposing. One useful benefit of these agents is the availability of comprehensive pharmacokinetic as well as safety data, which facilitates a quicker transition from preclinical research to clinical testing. Host kinase blockers have great potential, but their application as antivirals necessitates cautious safety evaluation. Numerous vital cellular functions are regulated by kinases, and their blockage can cause serious on-target toxicity, especially outside of oncologic settings or with extended treatment. Therefore, determining suitable dosage plans and treatment windows is still essential for their effective use in viral infections [69].

Since many viruses rely on particular lipid species for reproduction and assembly, manipulating lipid metabolism also presents antiviral prospects. Preclinical research has demonstrated the antiviral action of inhibitors of fatty acid synthase, acetyl-CoA carboxylase, and other metabolic enzymes. Toxicity issues are raised by the widespread significance of lipid metabolism [70,71]. Another tactic is the use of antagonists of host proteases, which are necessary for the digestion of viral proteins. Camostat mesylate has been studied as a COVID-19 treatment because it inhibits TMPRSS2, a serine protease that prepares the SARS-CoV-2 spike protein for entrance. Endosomal processing of viral proteins necessary for invasion can be prevented by cathepsin blockers [25].

The main issue for host-targeted antivirals is obtaining curative windows where antiviral impacts happen at levels below those that cause toxicity by interfering with normal cellular activities. Targeting host factors that are particular necessary during infection but unnecessary for normal cell function, concentrating medications at infection sites through tissue-specific delivery, and integrating host-targeted drugs with conventional antivirals to enable lower doses of each are some methods to enhance therapeutic windows [72]. High hurdles to resistance (viruses cannot readily modify host genes), possible broad-spectrum action against various viruses using the same host factors, and prospects for repurposing current medications are some benefits of host-targeted antivirals. Before virus-specific medications can be created, the timely production of host-targeted antivirals could give vital tools for pandemic preparedness, delivering alternatives potent against newly emerging viruses [73].

## 6. Emerging Technologies and Future Perspectives

Technology advancements that allow for previously unheard-of resolution and throughput in the research of viral infections are revolutionizing the science of host–virus relationships. These new technologies have the potential to expedite the identification of basic mechanisms and convert discoveries into medicinal uses. Study on antivirals and functional genomics has been revolutionized by CRISPR-Cas systems. Detailed maps of host–virus interactions are revealed by genome-wide CRISPR knockout screens, which methodically discover host genes necessary for or limiting viral infection. Several viruses, such as HIV-1, influenza, the Zika virus, and SARS-CoV-2, have undergone such screenings, which have shown both virus-specific dependence and retained host factors used by different viruses [74]. Functional investigations are made easier by CRISPR interference (CRISPRi) and activation (CRISPRa), which allow for reversible gene suppression or stimulation in addition to gene deletion. As shown by the SHERLOCK and DETECTR platforms used for COVID-19 testing, CRISPR-based diagnostics offer quick, efficient identification of viral nucleic acids [75]. CRISPR is additionally being studied for antiviral uses, such as focusing on viral genomes in infected cells to eradicate latent infections.

Bulk measurements obscure the variety in viral infection and host responses that single-cell approaches uncover. Only fractions of cells are actively infected, according to single-cell RNA sequencing of infected tissues, while nearby uninfected cells exhibit a variety of antiviral states. Infection dynamics, immunological responses, and treatment results are all impacted by this variability [76]. High-resolution tracing of gene functions in viral infection is made possible by single-cell CRISPR screens, which integrate single-cell readouts with individual gene perturbation [77]. By monitoring gene expression and maintaining tissue architecture, spatial transcriptomics reveals how infection moves throughout tissues and how local microenvironments affect results.

To give thorough understandings of host–virus connections, multi-omics techniques combine genomes, transcriptomics, proteomics, metabolomics, and other data types. Proteomics reveals processes not obvious from transcriptomics alone by identifying alterations in protein quantities, post-translational changes, and protein-protein relationships during infection. Phosphoproteomics identifies kinases and phosphorylation events that control viral replication or immune system reactions by mapping the activation of signaling pathways during infection. Metabolomics identifies possible metabolic bottlenecks by revealing how viruses rewire the metabolism of cells to facilitate reproduction. Systems-level knowledge of the infection can be obtained by integrating multi-omics data via computational methods [78].

The use of machine learning and artificial intelligence in host–virus interface research is growing. From extensive screening data, machine learning algorithms forecast host characteristics needed for viral multiplication. Deep learning uses image data analysis to measure physiological responses, protein localization, and viral infection at scale. In order to extract information regarding host–virus relationships and produce hypotheses, natural language processing searches scientific literature. By examining molecular structures, gene expression profiles, and network connections, AI-driven drug repurposing finds current medications with possible antiviral action. These computational methods speed up the creation of hypotheses and discoveries, but they need to be validated experimentally [79].

Compared to conventional cell culture, organoids and tissue engineering offer biologically applicable model systems that more accurately mimic human tissues. Investigations of viral tropism, pathogenicity, and host responses in human tissue contexts are made possible by lung, intestine, and brain organoids. These systems are especially useful for researching viruses that do not effectively infect traditional animal models and have limited host ranges. A variety of cells and tissue architecture are included into vascularized organoids and organ-on-a-chip systems to further improve their biological utility [80].

Host–virus interactions may be visualized with previously uncommon spatial and temporal resolution thanks to advanced imaging technology. Super-resolution microscopy reveals the organization of viral replication compartments and assembly sites by resolving subcellular structures below the diffraction limit. Ultrastructural detail and fluorescence imaging are combined in correlative light and electron microscopy. Using fluorescently labeled viral proteins, live-cell imaging monitors the changing patterns of infection in real time. By visualizing infection in living things, intravital microscopy offers new perspectives on immune reactions and tissue-level infection changes [81].

There are still a lot of obstacles and information gaps in spite of these technological advancements. Because host variables and immune responses vary by species, it is still challenging to translate results from cell culture and animal models to human infections. Acute infection models are the main focus of antiviral research, while persistent infections with complicated immunology and tissue pathology are more difficult to study experimentally. Although not fully understood, the interaction among viral infections and host variables such as nutrition, microbiota, genetics, and subsequent infections is crucial for predicting the course of individual diseases.

The development of more physiologically appropriate model systems that replicate the intricate structure of human tissue, the integration of multi-scale information from molecular to organismal levels, and the conversion of mechanistic discoveries into efficacious treatments and vaccines are among the future research goals. The COVID-19 pandemic has brought attention to the gaps in our readiness for new viral threats as well as the vital significance of comprehending host–virus interactions. Future pandemic preparation will depend on sustained funding for fundamental virology research, surveillance of possible pandemic infectious agents, and technological platforms for quick reaction.

## 7. Conclusions

Experimental research has shown that the specific involvement of host cell processes necessary for entry, intracellular transport, genome replication, and protein synthesis is intimately associated with viral replication and pathogenicity. It has been demonstrated that changes in membrane remodeling, endosomal trafficking, and protein quality control mechanisms affect the effectiveness of replication in a variety of viral families. Both viral limitation and tissue damage can result from the manipulation of host antiviral responses, which function within strictly controlled cellular networks during infection. The systematic identification of host reliance and restriction factors under specific experimental settings has been made possible by the recent use of functional genomic screens and proteomic analysis. These findings offer a mechanistic foundation for assessing immunomodulatory and host-specific therapies that target established cellular processes while maintaining crucial elements of normal cell function.

## Figures and Tables

**Table 1:** Molecular Interactions between host and virus.

Virus	Host Factor/Pathway	Molecular Interaction Mechanism	Functional Outcome	References
SARS-CoV-2	ACE2 receptor	Viral spike protein attaches ACE2 on host cell surface	Drives viral binding and entrance	[6]
SARS-CoV-2	TMPRSS2 protease	Host protease breaks spike protein	Allows membrane merge and access	[25]
HIV-1	NF-κB signaling	Viral Tat protein increases NF-κB–dependent transcription	Facilitates viral gene expression	[26]
Influenza A virus	Endosomal acidification	Reduced endosomal pH induces HA conformational shifts	Allows viral–endosomal membrane merge	[27]
Flaviviruses (DENV, ZIKV)	Endoplasmic reticulum membranes	Viral non-structural proteins reshapes ER membranes	Development of viral replication complexes	[28]
Herpesviruses	Nuclear pore complex	Capsid docking at nuclear pore for genetic material delivery	Creation of nuclear infection	[29]

## Data Availability

No new data were created or analyzed in this study.
